# Race- and Sex-Associated Electrocardiographic Repolarization Characteristics in Young American Athletes in the Digital Age

**DOI:** 10.1016/j.jacadv.2025.102409

**Published:** 2025-12-05

**Authors:** Jason V. Tso, Samuel Montalvo, Filipe Ferrari, Briana Collins, Skyler Jones, Jeffrey W. Christle, David Hadley, Bradley J. Petek, Victor Froelicher

**Affiliations:** a Division of Cardiovascular Medicine, Stanford Center for Inherited Cardiovascular Disease, Stanford, California, USA; b Division of Cardiovascular Medicine, Stanford University School of Medicine, Stanford, California, USA; c Graduate Program in Cardiology and Cardiovascular Sciences, School of Medicine, Universidade Federal do Rio Grande do Sul, Porto Alegre, Rio Grande do Sul, Brazil; d Department of Medicine, Stanford University School of Medicine, Stanford, California, USA; e Cardiac Insight Inc, Bellevue, Washington, USA; f Sports Cardiology Program, Knight Cardiovascular Institute, Oregon Health and Science University, Portland, Oregon, USA

**Keywords:** athletes, electrocardiography/ECG, screening, sports cardiology

## Abstract

**Background:**

The 2017 International Criteria for electrocardiographic (ECG) Interpretation in athletes include differential interpretation of anterior T-wave inversion (TWI) based on sex and race.

**Objectives:**

The purpose of this study was to analyze race- and sex-associated TWI differences and T-wave amplitudes in a digital ECG database of American athletes.

**Methods:**

We analyzed 8,758 athlete screening ECGs (mean age 18.5 ± 3.0 years; 58.7% male). TWI was defined as inversion <–0.075 mV (0.75 mm) to correlate with visual assessment. The convex anterior TWI pattern, historically attributed to Black athletes, was identified by J-point elevation >0.075 mV with convex ST morphology preceding TWI in >1 lead from V_2_ to V_4_.

**Results:**

TWI in ≥1 precordial lead (V_1_–V_6_) was present in 58% of athletes, and 7.5% had TWI in V_2_–V_4_. TWI was more common in females than males in V_1_ (80.6% vs 48.7%; *P* < 0.001), V_2_ (14.9% vs 4.4%; *P* < 0.001), and V_3_ (1.6% vs 0.3%; *P* < 0.001). Among males, TWI in V_4_-V_6_, II, and aVF was more common in Black than non-Black athletes. The convex anterior TWI pattern was rare overall (0.26%, n = 23), without sex- or race-associated differences (0.25% Black vs 0.27% non-Black athletes; *P* = 1.00). T-wave amplitude was associated with male sex and lower heart rate but not race.

**Conclusions:**

Anterior TWIs are common in young American athletes, with sex differences but minimal racial variation. The similar prevalence of the convex anterior TWI pattern suggests this pattern may be a normal finding in all races. Our findings offer digital reference values that may refine future ECG screening guidelines.

Preparticipation electrocardiographic (ECG) screening criteria for young athletes have evolved over the past several decades, with each iteration increasing specificity and cost-efficiency.^1^ A key factor in reducing false positive results was the reclassification of certain patterns of anterior T-wave inversion (TWI) as normal findings.^2^ The current International Criteria for ECG Interpretation in Athletes include race-specific criteria for normal precordial TWI: TWI in V_1_ and V_2_ is considered normal in all athletes, while TWI preceded by convex (domed) ST-segment elevation extending up to V_4_ is considered normal only in Black athletes.^3^

Anterior TWI is commonly observed in individuals with hypertrophic cardiomyopathy, acute myocardial ischemia, and arrhythmogenic right ventricular cardiomyopathy (ARVC).^4^ Several studies in European cohorts have shown that the convex anterior TWI pattern described above is present in more than 10% of Black athletes but is much less frequent among White athletes.^5^, ^6^, ^7^ These findings supported the consideration of race when evaluating anterior TWI in young athletes.^3^ It is important to note that race-specific ECG criteria persist despite contemporary views of race as a social construct with unreliable connection to biologic differences or genetic ancestry.^8^

This study aimed to analyze TWI patterns in a racially diverse population of American athletes, considering associations with sex and repolarization patterns. Based on our clinical experience, we hypothesized that race would not be associated with clinically significant differences in TWI, whereas sex would show a stronger association. To test this hypothesis, we conducted a comprehensive analysis of TWI using a large digital ECG database from young athletes undergoing preparticipation physical exams.

## Methods

We performed a retrospective analysis of a deidentified ECG preparticipation screening database of young competitive athletes, as previously described.^9^, ^10^, ^11^, ^12^, ^13^ Briefly, study sites across North America were selected based on their use of the Cardea 20/20 digital ECG system (Cardiac Insight Inc), a standardized hardware and software platform. All athletes underwent a digitally recorded 12-lead ECG during routine preparticipation evaluations.

This study included all athletes from 2014 to 2021, aged 14 to 35 years old without evidence of a baseline Wolff–Parkinson–White pattern, right or left bundle branch block, or reversed leads as all these scenarios may affect TWI patterns. Race was self-reported at the time of screening. The Stanford Institutional Review Board approved this study. Informed consent was obtained as required by local regulations, and athletes participated in standard cardiovascular screening per institutional guidelines. Since consent for follow-up was not possible for most athletes, data were deidentified, except in cases where investigators were responsible for clinical follow-up and consent for data use was obtained.

### Digital ECG processing and analysis

Although all modern ECG recorders digitize data according to established standards, most do not display average data or provide digital measurement outputs in an easily accessible format. The ECG system utilized across all sites extended the sampling window from the commonly used 10 seconds to 16 seconds to capture more beats, accommodating the slower heart rates of athletes. Digital ECG measurements were stored and made available in spreadsheet format.

### T-wave inversion measurements

To make digital criteria comparable to visual estimation, we adjusted our thresholds to account for the limitations of visual analysis by defining a lower cutoff for both TWI and ST-segment deviation, and by not requiring convex ST morphology to be present in contiguous leads to classify the convex anterior TWI pattern.

T waves were considered inverted if they had a negative value ≤−0.075 mV. The J-point was considered elevated if it exceeded 0.075 mV. A digital threshold of 0.075 mV was selected to correspond to 0.1 mV (equivalent to 1 mm at a 10 mm/mV scale) by visual estimation. For cases showing TWI and ST-segment elevation in leads V_2_-V_4_, 2 investigators (J.V.T., V.F.F.) reviewed the ECG tracings to confirm convex T-wave morphology.

To identify convex TWI, the following algorithm was used: (STI_Vx > ST_Vx) (ST_Vx >75) (T_Vx <−75), where *x* represents each respective precordial lead from V_1_ to V_4_, STI refers to the ST integral, ST to the ST-segment amplitude at the end of the QRS complex, and T to the T-wave amplitude.

### Statistical analysis

Analyses were performed using R (RStudio, version 4.5.0). Continuous variables are reported as mean ± SD, and normality was assessed using the Shapiro-Wilk test. Categorical variables are presented as counts and percentages.

### Prevalence comparisons

Lead-specific TWI rates were compared across sex, self-identified race (5 categories), and Black vs non-Black groups using chi-square or Fisher exact tests, as appropriate. Pairwise comparisons between racial groups were adjusted using the Bonferroni correction.

### Multivariate analysis

For each ECG lead, separate linear models were fitted for positive and negative T-wave amplitudes (dependent variable, in mV). Fixed effects included sex, race, age, body mass index (BMI), and resting heart rate. Model assumptions were verified using residual-vs.-fitted and Q-Q plots. Results are reported as β coefficients with 95% CIs. Statistical significance was set at α = 0.05 (2-tailed).

## Results

A total of 8,758/10,728 (82%) athletes met the inclusion criteria (3,614 females [41.3%], 5,144 males [58.7%]), with a mean age of 18.5 ± 3.0 years. Mean weight was 73 ± 19 kg, mean height 175.1 ± 11.4 cm, and mean BMI 23.5 ± 4.2 kg/m^2^. We excluded individuals with Wolff–Parkinson–White pattern (n = 26), right or left bundle branch block (n = 77), or reversed leads (n = 35), and included only those aged 14 to 35 years. The self-reported racial/ethnic composition was as follows: 1,213 Black (14%), 760 Asian (8.7%), 5,454 White (62%), 744 Hispanic (8.5%), and 587 (6.7%) identifying as other (Table 1).

**Table 1:** Overall Demographic Characteristics of the Athlete Cohort

	Overall (N = 8,758)	Female (n = 3,614)	Male (n = 5,144)	*P* Value^a^
Age (y)	18.49 (2.98)	18.04 (2.23)	18.81 (3.38)	<0.001
Weight (kg)	73 (19)	63 (12)	81 (20)	<0.001
Height (cm)	175.1 (11.4)	167.1 (8.4)	180.8 (9.6)	<0.001
Body mass index	23.5 (4.2)	22.4 (3.4)	24.4 (4.5)	<0.001
Resting heart rate (beats/min)	65 (12)	66 (13)	64 (12)	<0.001
Race/Ethnicity				<0.001
Black	1,213 (14%)	306 (8.5%)	907 (18%)	
Asian	760 (8.7%)	358 (9.9%)	402 (7.8%)	
White	5,454 (62%)	2,363 (65%)	3,089 (60%)	
Hispanic	744 (8.5%)	321 (8.9%)	425 (8.3%)	
Other	587 (6.7%)	266 (7.4%)	321 (6.2%)	
Class				<0.001
College	5,168 (59%)	2,347 (65%)	2,821 (55%)	
Grade school	99 (1.1%)	38 (1.1%)	61 (1.2%)	
High school	2,928 (33%)	1,197 (33%)	1,731 (34%)	
Professional	556 (6.4%)	29 (0.8%)	527 (10%)	

a Wilcoxon rank sum test; Pearson’s chi-squared test.

### T-wave inversion

**Figure 1:**
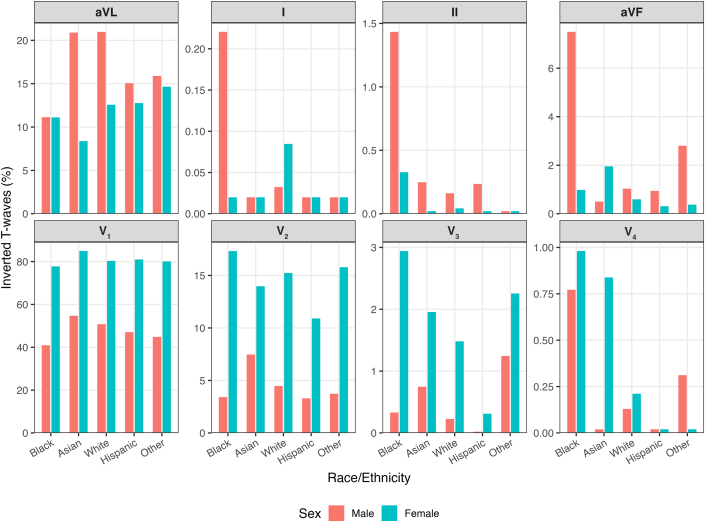
**Proportion of Inverted T Waves in Each ECG Lead by Race and Sex** ECG = electrocardiogram/electrocardiographic.

Overall, 58.0% of athletes demonstrated TWI in at least 1 precordial lead (V_1_–V_6_), and 7.5% demonstrated TWI in one or more of leads V_2_–V_4_. Negative precordial TWI beyond V_2_ was very uncommon in males (<0.3%). Among females, TWI in V_3_ was present in 1.6% but very uncommon beyond V_4_ (<0.3%) (Figure 1, Table 2).

**Table 2:** Counts and Percentages of Male and Female Athletes With Negative Precordial and Limb T Waves

Lead	Male (n = 5,144)	Female (n = 3,614)	*P* Value
V_1_	2,504 (48.7%)	2,913 (80.6%)	<0.001
V_2_	225 (4.4%)	540 (14.9%)	<0.001
V_3_	17 (0.3%)	58 (1.6%)	<0.001
V_4_	12 (0.2%)	11 (0.3%)	0.325
V_5_	13 (0.3%)	1 (0%)	0.011
V_6_	14 (0.3%)	1 (0%)	0.007
aVL	948 (18.4%)	441 (12.2%)	<0.001
I	3 (0.1%)	2 (0.1%)	1.000
II	20 (0.4%)	2 (0.1%)	0.002
aVF	115 (2.2%)	26 (0.7%)	<0.001
III	1,000 (19.4%)	637 (17.6%)	0.032

Female athletes had a higher prevalence of precordial TWI than males in leads V_1_ (80.6% vs 48.7%; *P* < 0.001), V_2_ (14.9% vs 4.4%; *P* < 0.001), and V_3_ (1.6% vs 0.3%; *P* < 0.001), whereas no sex difference was seen in V_4_ (0.3% vs 0.2%; *P* = 0.532). In contrast, males exhibited modestly higher rates than females in V_5_ (0.3% vs 0.0%; *P* = 0.011) and V_6_ (0.3% vs 0.0%; *P* = 0.007) (Table 2). Among female athletes, Black and non-Black athletes showed similar TWI prevalence across all precordial leads. Conversely, Black male athletes had significantly greater TWI than non-Black males in leads V_4_, V_5_, V_6_, II, and aVF (Figure 1; Supplemental Table 1). In the limb leads, males demonstrated higher TWI rates than females in aVL (18.4% vs 12.2%; *P* < 0.001), II (0.4% vs 0.1%; *P* < 0.002), aVF (2.2% vs 0.7%; *P* < 0.001), and III (19.4% vs 17.6%; *P* = 0.032) (Table 2). Note that TWI in aVL is common (approximately 1 in 8 male athletes and ∼12% of females) making it relevant as a likely benign variant.

### T-wave inversion by race

In lead V_2_, TWI was less frequent in Black athletes than in non-Black athletes (6.9% vs 9.2%; *P* = 0.019), whereas rates were similar in lead V_3_ (1.0% vs 0.9%; *P* = 0.709). Black athletes exhibited a higher prevalence of TWI in leads V_4_ (0.82% vs 0.17%; *P* < 0.001), V_5_ (0.66% vs 0.16%; *P* < 0.001), V6 (0.66% vs 0.17%; *P* < 0.001), II (1.15% vs 0.25%; *P* < 0.001), and aVF (5.85% vs 1.61%; *P* < 0.001) compared to non-Black athletes (Table 3).

**Table 3:** T-Wave Inversion Prevalence by Race

Lead	Overall (%)	Black (%)	White (%)	Asian (%)	Hispanic (%)	Other (%)	*P* Value	
							Overall	Black vs Non-Black
V_1_	61.85	50.21	63.59	68.95	61.66	60.82	<0.001	<0.001
V_2_	8.73	6.92	9.13	10.53	6.57	9.20	0.01	0.019
V_3_	0.85	0.99	0.77	1.32	0.13	1.70	0.02	0.70
V_4_	0.26	0.82	0.16	0.40	0.00	0.17	0.001	<0.001
V_5_	0.16	0.66	0.09	0.00	0.13	0.00	<0.001	<0.001
V_6_	0.17	0.66	0.11	0.00	0.13	0.00	<0.001	<0.001
II	0.25	1.15	0.11	0.13	0.13	0.00	<0.001	<0.001
III	18.69	32.40	15.84	18.68	17.69	18.06	<0.001	<0.001
aVF	1.61	5.85	0.84	1.18	0.67	1.70	<0.001	<0.001
I	0.05	0.17	0.05	0.00	0.00	0.00	0.40	0.30
aVL	15.86	11.13	17.33	15.00	14.07	15.33	<0.001	<0.001

### Convex anterior T-wave inversion

Overall, convex TWI in any lead from V_1_ to V_4_ was uncommon in both sexes but more frequent in males. Among females, prevalence ranged from 0.25% in White athletes to 0.65% in Black athletes, with only leads V_1_ and V_2_ exceeding 0.5% in Asian and Hispanic athletes. Among males, rates were higher—peaking at 1.98% in Black athletes and 1.74% in Asian athletes—with most convex TWI limited to V_1_–V_2_. White and “other” males had lower prevalences (0.58% and 0.93%, respectively) (Table 4).

**Table 4:** Prevalence of Convex T-Wave Inversion in the Anterior Leads (V_1_–V_4_)

Sex	Race	Any (%)	V_1_ (%)	V_2_ (%)	V_3_ (%)	V_4_ (%)	V_3_ and/orV_4_ (%)
Female	Black	0.65	0.33	0.00	0.33	0.00	0.33
Female	Asian	0.56	0.56	0.00	0.00	0.00	0.00
Female	White	0.25	0.04	0.17	0.00	0.04	0.04
Female	Hispanic	0.62	0.00	0.62	0.00	0.00	0.00
Female	Other	0.38	0.00	0.38	0.00	0.00	0.00
Male	Black	1.98	0.88	0.55	0.22	0.33	0.55
Male	Asian	1.74	1.24	0.50	0.00	0.00	0.00
Male	White	0.58	0.36	0.16	0.10	0.03	0.13
Male	Hispanic	1.41	1.18	0.24	0.00	0.00	0.00
Male	Other	0.93	0.00	0.62	0.31	0.31	0.62

### Convex anterior T-wave inversion extending beyond V_2_

**Figure 2:**
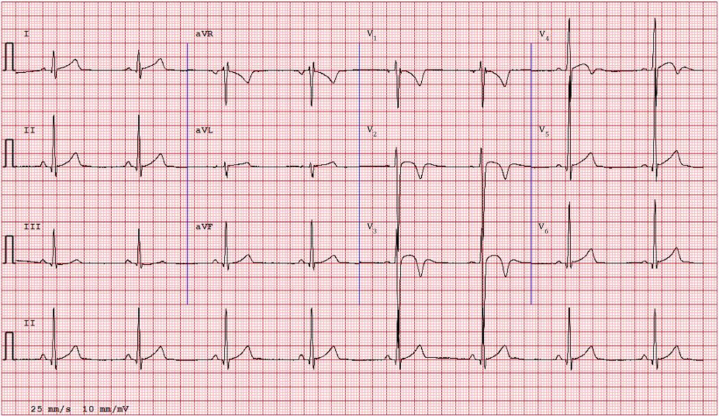
**“Black Athlete Repolarization Pattern” With J-Point Elevation, Convex ST-Segment, and T-Wave Inversion** This example is from an 18-year-old White cross-country runner whose brother had a similar pattern. The pattern normalized when the athlete was injured and detrained. The same pattern was also observed in 2 Hispanic sisters who also were cross-country runners.

**Figure 3:**
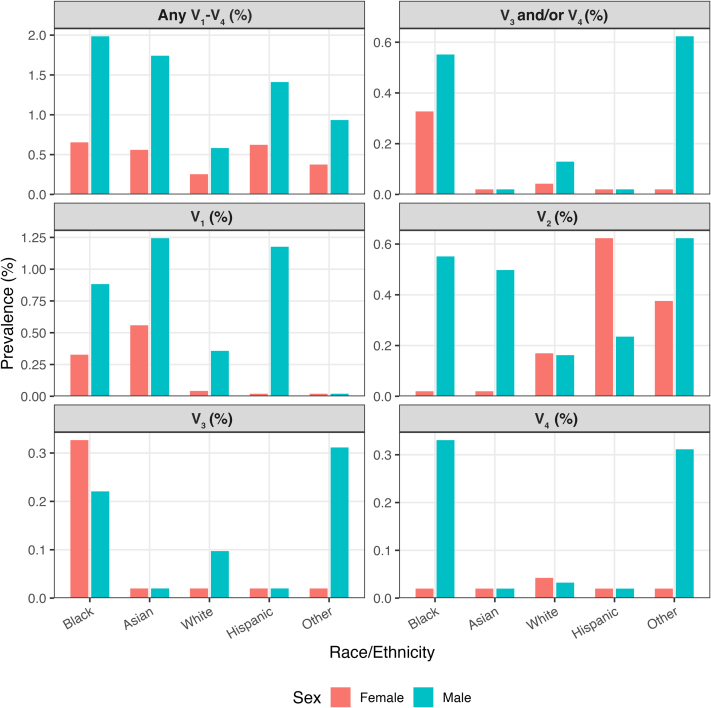
Percentage of Participants Exhibiting a Convex Anterior T-Wave Inversion Pattern Across Race/Ethnicity in Leads V_1_-V_4_, Shown Separately for Females and Males.

Convex anterior TWI extending beyond V_2_, previously termed the “Black athlete repolarization variant,” was rare (<1% prevalence) across all demographic subgroups (Figures 2 and 3; Central Illustration). Overall, the pattern was observed in only 23 athletes (0.26% of the cohort). There was no significant difference in prevalence between racial groups (overall *P* = 0.90; Black vs non-Black; *P* = 1.00). With n_1_ = 1,213 Black and n_2_ = 7,545 non-Black (baseline 0.49%), the minimal detectable absolute difference at 80% power is 0.79 percentage points (2-sided α = 0.05), indicating adequate power for ≥0.79 percentage points differences but limited sensitivity to smaller race gaps. Among Black athletes, it occurred in 3 of 1,213 (0.25%); among Asian athletes, in 3 of 760 (0.40%); among White athletes, in 13 of 5,452 (0.24%); among Hispanic athletes, in 3 of 746 (0.40%); and among ‘other” athletes, in 2 of 587 (0.34%). When stratified by sex, it was seen in 11 of 3,614 females (0.30%) and 12 of 5,144 males (0.23%).

**Central Illustration:**
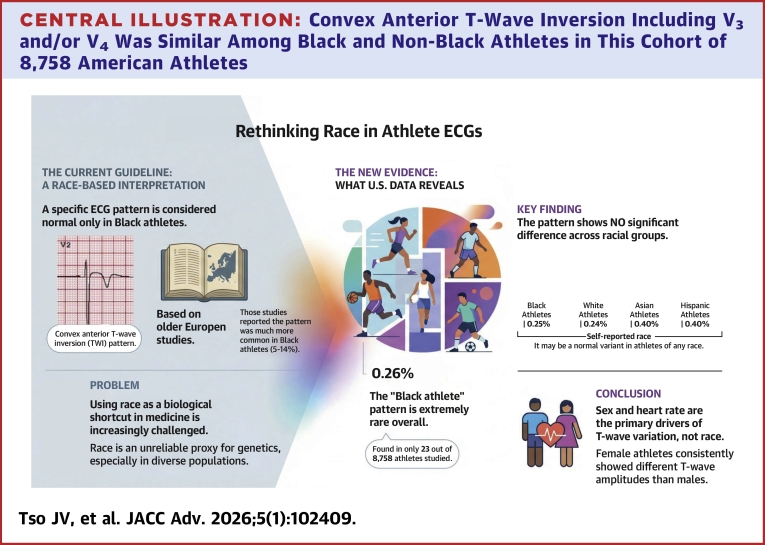
**Convex Anterior T-Wave Inversion Including V_3_ and/or V_4_ Was Similar Among Black and Non-Black Athletes in This Cohort of 8,758 American Athletes** Race was not associated with the athlete anterior repolarization pattern or T-wave amplitude.

### Determining high-risk enhanced criteria

**Figure 4:**
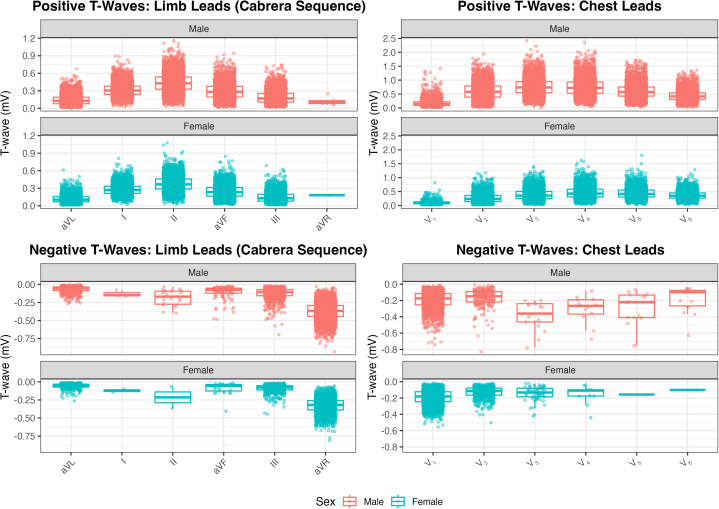
**Boxplot With Jitter of T-Wave Amplitude (mV)** Top panels: positive waves; bottom panels: negative waves.

The full distributions of positive and negative T-wave amplitudes are shown in Figure 4, enabling visual identification of outliers. The same data were used to calculate empiric 98th and 99th percentile cutoffs for each lead, stratified by sex and polarity (Table 5). These cutoffs illustrate the extreme ends of normal T-wave amplitude distributions within this athletic population and may serve as benchmarks for future research or refinement of screening guidelines.^10^ Importantly, amplitudes exceeding these limits do not necessarily indicate underlying cardiac pathology. Instead, they represent values beyond typical variation and should be interpreted in conjunction with clinical history, imaging, and other diagnostic findings.

**Table 5:** The 98th/99th Percentiles for Each Lead Amplitude (mV) Represented by Sex, Lead, and T-Wave Polarity Providing Potential Enhanced Criteria for Identifying Subjects at Risk of Cardiac Disease

Sex	Sign	V_1_	V_2_	V_3_	V_4_	V_5_	V_6_
Precordial Leads							
Male	+TW	0.56|0.67	1.25|1.36	1.43|1.56	1.46|1.61	1.24|1.38	0.90|1.02
Male	-TW	−0.05|−0.04	−0.03|−0.02	−0.20|−0.19	−0.10|−0.09	−0.08|−0.07	−0.05|−0.05
Female	+TW	0.38|0.43	0.72|0.78	0.88|0.99	1.00|1.09	0.91|0.99	0.72|0.80
Female	-TW	−0.05|−0.04	−0.03|−0.03	−0.030|−0.02	−0.04|−0.04	−0.15|0.16	−0.10|−0.10

**Sex**	**Sign**	**aVL**	**I**	**II**	**aVF**	**III**	**aVR**

Limb Leads							
Male	+TW	0.36|0.41	0.57|0.62	0.78|0.84	0.60|0.66	0.47|0.53	0.24|0.24
Male	-TW	−0.02|−0.01	−0.08|−0.08	−0.05|−0.04	−0.028|−0.02	−0.030|−0.02	−0.15|−0.13
Female	+TW	0.29|0.34	0.51|0.54	0.66|0.70	0.50|0.54	0.38|0.42	0.18|0.18
Female	-TW	−0.02|−0.01	−0.10|−0.09	−0.07|−0.06	−0.02|−0.01	−0.02|−0.02	−0.15|−0.12

### Multivariate analysis

Twenty-four models were constructed (12 leads, 2 polarities) using sex, race, age, BMI, and resting heart rate as predictors. Across all models, female sex was the strongest and most consistent determinant of T-wave amplitude. In the negative-wave models, female sex was associated with smaller (less negative) amplitudes—for example, 198 μV smaller in V_3_ and 42.8 μV smaller in aVR (all *P* < 0.001). In positive-wave models, female sex predicted lower T-wave peaks, ranging from 22.8 μV smaller in lead I to 379.5 μV smaller in lead V_3_ (all *P* < 0.001).

**Figure 5:**
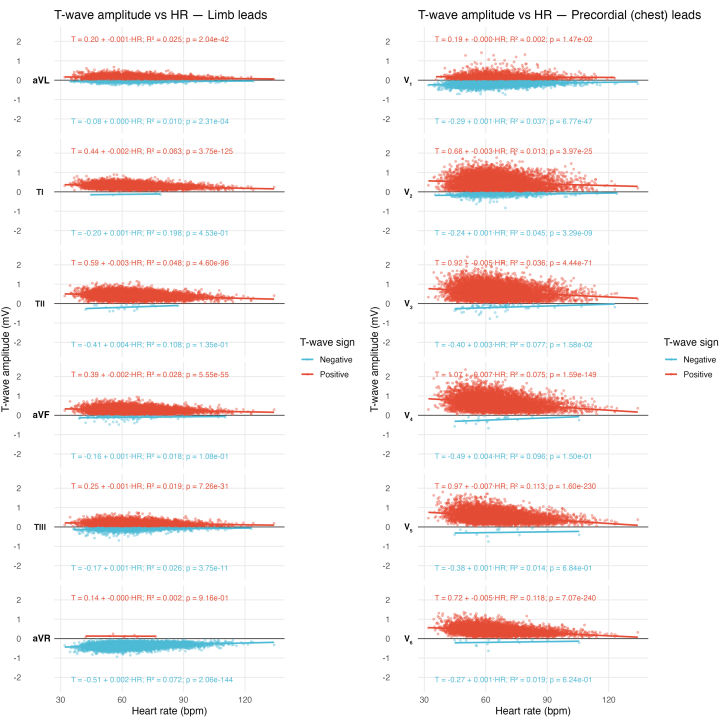
Increased Resting Heart Rate Is Associated With Lower T-Wave Amplitude

The sex difference was especially pronounced in the precordial leads, with average absolute female T-wave voltage reduced by −0.35 mV in V_2_, –0.38 mV in V_3_, and –0.30 mV in V_4_, compared to males. Smaller but still significant differences were also seen in limb leads (eg, −0.03 mV in lead I, −0.07 mV in lead II). Resting heart rate emerged as the second most consistent correlate: each 1 beats/min increase was associated with smaller T-wave shifts in negative-wave models (+0.7 to +2.5 μV per beats/min) and smaller peaks in the positive-wave models (−0.2 to −6.4 μV per beats/min) (all *P* < 0.001) (Figure 5, Supplemental Table 2).

By contrast, age had minimal clinical relevance, with coefficients typically <6 μV per year or near zero, and BMI effects—though statistically significant in some leads—were uniformly small (absolute changes ≤6 μV per BMI unit, or ±0.01 mV), suggesting limited impact.

Race did not show a consistent or meaningful association with T-wave amplitude in any model. Sex and heart rate were the primary drivers of T-wave amplitude variation in young athletes, while age, BMI, and race had minimal influence.

## Discussion

This study analyzed repolarization patterns in a large cohort of young American athletes, with a focus on identifying race- and sex-associated differences. Key findings are as follows. First, while anterior TWI has been proposed as a marker of athletic ECG remodeling in athletes, only 77 out of 8,758 (0.88%) of athletes in our cohort demonstrated anterior TWI in V_3_ and/or V_4_. Second, precordial TWI extending to V_3_ was more common in females than males and race-associated differences in TWI were only observed among male athletes, specifically in leads V_4_, V_5_, and V_6_. Third, the convex anterior TWI pattern—historically linked to Black athletes—was rare (<0.5%) and showed no meaningful racial predilection in either sex. Collectively, these findings suggest that, although subtle race-associated differences in repolarization exist, the clinical utility of race-based ECG screening criteria may be limited in young American athletes.

The International Criteria recognize anterior TWI as a common ECG finding in hypertrophic cardiomyopathy and ARVC, but an exception is made for Black athletes when convex ST morphology precedes TWI—a pattern described more frequently in this group.^3^ The evidence supporting this exception is largely based on European cohorts, where anterior TWI has been reported in 5% to 14% of Black athletes compared to 0% to 4% of White athletes^5^^,^^6^^,^^14^^,^^15^ (Table 6). However, data from non-European cohorts show substantially lower rates. For example, only 2.6% of young Black professional American football athletes had abnormal TWI (including, but not restricted to anterior leads), and just 1.8% of Black Brazilian soccer players demonstrated the convex pattern.^16^^,^^17^ This may suggest that North and South American athletes are of more diverse ethnic background, resulting in more mixed race individuals identifying with a single race category. These intercontinental discrepancies call into question the generalizability of ECG norms based on categorical race classifications.

**Table 6:** Select Studies Evaluating Prevalence of Anterior T-Wave Inversion in Athletes

First Author. *Journal*, Year	Prevalence of Anterior T-Wave Inversion in Black/White Athletes	Definition of Anterior T-Wave Inversion	Athlete Population
Papadakis et al. *Eur Heart J*, 2011^6^	Black athletes: 12.7% White athletes: 1.9%	T-wave inversion confined to the anterior leads (V_1_–V_4_)	2,723 athletes from the United Kingdom and France (904 Black, 1,819 White)
Zaidi et al. *Circulation*, 2013^15^	Black athletes: 14.3% (81% with preceding convex ST segments) White athletes: 3.7% Black controls: 5.8% White controls: 2.4%	T-wave inversion >1 mm in ≥2 contiguous in V_1_ to V_4_	300 Black athletes, 375 White Athletes, 153 sedentary controls from the United Kingdom
Malhotra et al. *J Am Coll Cardiol*, 2017^5^	Female athletes: 6.5% (2.1% beyond V_2_) Male athletes: 2.5% (0.3% beyond V_2_)	T-wave inversion in ≥2 contiguous anterior leads (V_1_ to V_4_)	14,646 White United Kingdom athletes (2,958 athletes)
Magalski et al. *J Am Coll Cardiol*, 2008^16^	Black athletes: 2.6% White athletes: 0.2%	Any leads: >2 mm T-wave inversion in >2 leads	1,959 professional American football combine participants
Ferrari et al. *Eur J Prev Cardiol* 2024^17^	Afro-Caribbean pattern: Black athletes: 1.8% Mixed-race athletes: 0.3%	T-wave inversion only, no ST analysis	1,627 Black and 2,004 mixed-race Brazilian soccer players
Riding et al. *Eur Heart J*, 2019^18^	African American/Caribbean: 3.3% East African: 2.0% Middle African: 13.5% West African: 7.8% South American: 3.5% West Asian: 3.4% North African: 0.7% South European White: 0%	T-wave inversion >1 mm in ≥2 contiguous in V_2_ to V_4_	1,013 Black athletes from various geographic regions, 418 non-Black North African/Arabic athletes, 267 South European White athletes
Sheikh et al. *Br J Sports Med*, 2013^19^	TWI V_1_-V_4_: Black athletes: 14.3% (3.6% confined to V_1_-V_2_) Black controls: 11.9% (10.4% confined to V_1_-V_2_) White athletes: 2.5% (2.3% confined to V_1_-V_2_) Deep TWI: 6.7% Black athletes 0.2% White athletes 0.7% Black controls	T-wave inversion >0.1 mV in >2 leads Deep T-wave inversion: >0.2 mV	329 Black athletes, 134 Black controls, 903 White athletes from the UK and France
Rim et al. *JACC Adv*, 2025^20^	Black athletes: 1.91% Non-Black athletes: 0.48%	Convex ST-elevation preceding anterior T-wave inversion extending to V_3_ or V_4_	1,203 Black athletes, 4,974 non-Black athletes from multiple centers in the United States

While the original classification of the convex pattern as a benign variant in the “Black athlete’s heart” was intended to reduce false positive results in Black athletes,^3^ it may also imply biologically deterministic interpretations of racial differences. Contemporary perspectives in medicine increasingly emphasize the complex interplay of social, environmental, and historical factors that shape race-associated health patterns.^8^^,^^21^ In the United States, self-described race is an unreliable proxy for genetic ancestry due to extensive admixture and regional heterogeneity.^22^ Even among African athletes, anterior TWI prevalence ranges from 2% to 13% depending on geographic region.^18^ Our findings—showing minimal race-associated differences in anterior repolarization patterns—further challenge rigid phenotypic categorization based on race.

In a recent study of American athletes by Rim et al, investigators studied over 6,000 ECGs from 4 centers, including 19% self-reported Black athletes.^20^ In their study, 0.48% of White athletes displayed ascending convex ST segments with TWI in the anterior leads, compared to 1.9% of Black athletes, consistent with our findings. Their study utilized traditional visual ECG interpretation, while our digital analysis provides precise quantification of T-wave amplitudes. Together, these studies complement each other: Rim et al offer careful visual validation, and our work demonstrates the utility of computerized ECG T-wave analysis in athletes.

Taken together, the lack of consistent race-associated differences may support the reclassification of the convex TWI pattern as a normal variant in athletes, regardless of race. A longitudinal study of athletes with anterior TWI followed over 8.3 years found no subsequent development of hypertrophic cardiomyopathy or ARVC,^19^ and a genetic study of 100 Black and White athletes with isolated anterior TWI revealed no structural abnormalities or pathogenic variants.^14^ Given these data, we suggest that the convex TWI pattern on screening ECG may be a physiological adaptation rather than a racial phenotype, provided athletes are asymptomatic and have no concerning family history.^23^ This distinction is crucial, as the convex pattern resembles repolarization changes seen in hypertrophic cardiomyopathy, ARVC, and acute ischemia.

### Clinical application of outliers

Care must be taken with the use of outliers as they may be caused by data artifacts or a sign of early influences of pathology.^24^ The raw ECG from candidate outliers must always be examined to confirm that signal processing was not affected by artifact as we have done in our methodology. Outliers should not be assumed as identifying pathology but may be considered when subjects have worrisome symptoms, clinical/familial history, or other abnormalities (enzyme elevations, borderline genetic, or test results). Their use provides the opportunity to increase sensitivity without much degradation of specificity (Table 5).

### The digital age

Traditional ECG interpretation from paper tracings has inherent limitations-beat-to-beat variability, respiratory influence, and lead axis shifts—that are mitigated by digital averaging techniques.^24^ Standard clinical tracings present just 2.5 seconds per lead, capturing at most 2 cardiac cycles, whereas digital ECG analyzes 10 or more beats over 10 seconds. Manual readings tend to highlight the most abnormal beat and rely on visual estimates (rounded to the nearest 1 mm), introducing measurement bias. Moreover, abnormalities in adjacent leads can be conflated due to perceptual “crosstalk.” To improve fidelity in our digital analysis, we employed amplitude thresholds below the standard 1 mm cutoffs and did not require contiguous convex ST morphology to identify the convex TWI pattern.

### Study limitations

The primary limitation of this study is the absence of echocardiographic or clinical follow-up. Thus, the assumption that athletes with the convex anterior TWI pattern did not harbor occult pathology relies on prior reports, and should be interpreted cautiously.^6^^,^^23^^,^^25^ Second, while the most athletes self-identified as White, our cohort remains more racially diverse that prior studies and, to our knowledge, is the largest to date examining ECG repolarization differences in a racially heterogeneous American athlete population. Finally, the use of self-identified race—an imprecise and nominal classification—is a methodological limitation common to nearly all investigations of race and cardiovascular phenotypes. Unfortunately, our Institutional Review Board protocol did not permit longitudinal follow-up, and as such, only approximately half of our athlete cohort received scanning echocardiograms or clinical follow-up, primarily those seen at Stanford. We have had a low event rate observed in our study, which we attribute to the comprehensive medical care received by our study population prior to sports participation, a reflection of existing health care disparities in the United States.

## Conclusions

In this large and racially diverse cohort of young American athletes, anterior TWI beyond V_2_ was rare, and the coving/convex ST-segment pattern—historically associated with Black athletes—was uncommon and present across all racial groups. Sex and heart rate were the primary determinants of T-wave amplitude, while race had minimal influence after adjustment. These findings challenge the clinical relevance of race-based distinctions in ECG interpretation among young competitive athletes and may support the recognition of convex anterior TWI as a normal variant in athletes, irrespective of race. Further longitudinal outcome studies are needed to confirm this finding. Our study also provides digital measurements that should be considered by future screening guidelines to increase sensitivity without significantly compromising specificity.

## Funding support and author disclosures

Dr Hadley, now retired, was previously employed by Cardiac Insight Inc. Dr Froelicher is the chief medical officer of Cardiac Insight Inc. All other authors have reported that they have no relationships relevant to the contents of this paper to disclose.
