# Supplementary material for: Race- and Sex-Associated Electrocardiographic Repolarization Characteristics in Young American Athletes in the Digital Age
Source: JACC Adv. 2025 Dec 5;5(1):102409. doi: 10.1016/j.jacadv.2025.102409 (PMC12723155; doi:10.1016/j.jacadv.2025.102409)
Supplement: Supplemental Table 1 and 2 [file mmc1.docx]

**Supplemental Table 1. Sex-stratified T-wave Inversion Prevalence**

| **Lead** | **Overall (%)** | **Black (%)** | **White (%)** | **Asian (%)** | **Hispanic (%)** | **Other (%)** | **p-value (Overall)** | **p-value (Black vs. Non-Black)** | **Sex** |
| --- | --- | --- | --- | --- | --- | --- | --- | --- | --- |
| V1 | 80.71 | 77.78 | 80.47 | 84.92 | 81.19 | 80.08 | 0.20 | 0.20 | Female |
| V2 | 14.97 | 17.32 | 15.35 | 13.97 | 10.34 | 15.79 | 0.12 | 0.26 | Female |
| V3 | 1.63 | 2.94 | 1.48 | 1.96 | 0.63 | 2.26 | 0.17 | 0.10 | Female |
| V4 | 0.30 | 0.98 | 0.21 | 0.84 | 0.00 | 0.00 | 0.03 | 0.09 | Female |
| V5 | 0.03 | 0.00 | 0.04 | 0.00 | 0.00 | 0.00 | 0.97 | 1.00 | Female |
| V6 | 0.03 | 0.00 | 0.04 | 0.00 | 0.00 | 0.00 | 0.97 | 1.00 | Female |
| II | 0.06 | 0.33 | 0.04 | 0.00 | 0.00 | 0.00 | 0.32 | 0.40 | Female |
| III | 17.68 | 23.86 | 15.90 | 24.02 | 18.18 | 17.29 | 0.00 | 0.00 | Female |
| aVF | 0.72 | 0.98 | 0.59 | 1.96 | 0.31 | 0.38 | 0.05 | 0.83 | Female |
| I | 0.06 | 0.00 | 0.08 | 0.00 | 0.00 | 0.00 | 0.90 | 1.00 | Female |
| aVL | 12.23 | 11.11 | 12.60 | 8.38 | 12.85 | 14.66 | 0.13 | 0.59 | Female |
| V1 | 48.78 | 40.90 | 50.96 | 54.73 | 47.06 | 44.86 | 0.00 | 0.00 | Male |
| V2 | 4.39 | 3.42 | 4.50 | 7.46 | 3.29 | 3.74 | 0.01 | 0.14 | Male |
| V3 | 0.33 | 0.33 | 0.23 | 0.75 | 0.00 | 1.25 | 0.01 | 1.00 | Male |
| V4 | 0.23 | 0.77 | 0.13 | 0.00 | 0.00 | 0.31 | 0.01 | 0.00 | Male |
| V5 | 0.25 | 0.88 | 0.13 | 0.00 | 0.24 | 0.00 | 0.00 | 0.00 | Male |
| V6 | 0.27 | 0.88 | 0.16 | 0.00 | 0.24 | 0.00 | 0.00 | 0.00 | Male |
| II | 0.39 | 1.43 | 0.16 | 0.25 | 0.24 | 0.00 | 0.00 | 0.00 | Male |
| III | 19.46 | 35.28 | 15.86 | 13.93 | 17.65 | 18.69 | 0.00 | 0.00 | Male |
| aVF | 2.24 | 7.50 | 1.04 | 0.50 | 0.94 | 2.80 | 0.00 | 0.00 | Male |
| I | 0.06 | 0.22 | 0.03 | 0.00 | 0.00 | 0.00 | 0.28 | 0.14 | Male |
| aVL | 18.47 | 11.14 | 21.04 | 20.90 | 15.06 | 15.89 | 0.00 | 0.00 | Male |

**Supplemental Table 2** – Heart rate and T-wave amplitude in each ECG lead

| **Lead** | **Sex** | **T-wave sign** | **n** | **Intercept (mV)** | **Slope (mV/bpm)** | **R²** | **p-value** |
| --- | --- | --- | --- | --- | --- | --- | --- |
| TI | Female | Negative | 2 | NA | NA | NA | NA |
| TI | Female | Positive | 3608 | 0.401 | -0.002 | 0.062 | 5.68e-52 |
| TI | Male | Negative | 3 | -0.280 | 0.003 | 0.775 | 3.14e-01 |
| TI | Male | Positive | 5134 | 0.454 | -0.002 | 0.057 | 2.14e-67 |
| TII | Female | Negative | 2 | NA | NA | NA | NA |
| TII | Female | Positive | 3608 | 0.551 | -0.003 | 0.065 | 1.61e-54 |
| TII | Male | Negative | 20 | -0.395 | 0.003 | 0.116 | 1.42e-01 |
| TII | Male | Positive | 5117 | 0.591 | -0.002 | 0.034 | 1.62e-40 |
| TIII | Female | Negative | 637 | -0.118 | 0.000 | 0.010 | 1.20e-02 |
| TIII | Female | Positive | 2973 | 0.212 | -0.001 | 0.018 | 3.17e-13 |
| TIII | Male | Negative | 1000 | -0.189 | 0.001 | 0.022 | 1.93e-06 |
| TIII | Male | Positive | 4137 | 0.264 | -0.001 | 0.016 | 7.54e-16 |
| TV1 | Female | Negative | 2913 | -0.293 | 0.002 | 0.051 | 6.97e-35 |
| TV1 | Female | Positive | 697 | 0.122 | 0.000 | 0.001 | 4.39e-01 |
| TV1 | Male | Negative | 2504 | -0.282 | 0.001 | 0.026 | 3.52e-16 |
| TV1 | Male | Positive | 2633 | 0.193 | 0.000 | 0.000 | 3.53e-01 |
| TV2 | Female | Negative | 540 | -0.224 | 0.001 | 0.062 | 4.06e-09 |
| TV2 | Female | Positive | 3070 | 0.348 | -0.001 | 0.008 | 9.54e-07 |
| TV2 | Male | Negative | 225 | -0.262 | 0.001 | 0.032 | 7.54e-03 |
| TV2 | Male | Positive | 4912 | 0.733 | -0.002 | 0.007 | 3.38e-09 |
| TV3 | Female | Negative | 58 | -0.247 | 0.002 | 0.061 | 6.26e-02 |
| TV3 | Female | Positive | 3552 | 0.570 | -0.003 | 0.030 | 2.08e-25 |
| TV3 | Male | Negative | 17 | -0.475 | 0.001 | 0.010 | 7.01e-01 |
| TV3 | Male | Positive | 5120 | 1.048 | -0.004 | 0.034 | 1.01e-40 |
| TV4 | Female | Negative | 11 | -0.427 | 0.004 | 0.073 | 4.20e-01 |
| TV4 | Female | Positive | 3599 | 0.755 | -0.004 | 0.071 | 1.19e-59 |
| TV4 | Male | Negative | 12 | -0.647 | 0.005 | 0.245 | 1.02e-01 |
| TV4 | Male | Positive | 5125 | 1.194 | -0.007 | 0.076 | 2.12e-90 |
| TV5 | Female | Negative | 1 | NA | NA | NA | NA |
| TV5 | Female | Positive | 3609 | 0.787 | -0.005 | 0.124 | 1.58e-105 |
| TV5 | Male | Negative | 13 | -0.390 | 0.001 | 0.016 | 6.80e-01 |
| TV5 | Male | Positive | 5124 | 1.041 | -0.007 | 0.106 | 1.41e-126 |
| TV6 | Female | Negative | 1 | NA | NA | NA | NA |
| TV6 | Female | Positive | 3609 | 0.649 | -0.004 | 0.137 | 1.95e-117 |
| TV6 | Male | Negative | 14 | -0.280 | 0.001 | 0.020 | 6.32e-01 |
| TV6 | Male | Positive | 5123 | 0.755 | -0.005 | 0.102 | 3.32e-122 |
| TaVF | Female | Negative | 26 | -0.113 | 0.000 | 0.004 | 7.69e-01 |
| TaVF | Female | Positive | 3584 | 0.357 | -0.002 | 0.037 | 1.79e-31 |
| TaVF | Male | Negative | 115 | -0.167 | 0.001 | 0.018 | 1.54e-01 |
| TaVF | Male | Positive | 5022 | 0.391 | -0.002 | 0.019 | 2.81e-22 |
| TaVL | Female | Negative | 441 | -0.068 | 0.000 | 0.004 | 1.81e-01 |
| TaVL | Female | Positive | 3169 | 0.164 | -0.001 | 0.016 | 5.50e-13 |
| TaVL | Male | Negative | 948 | -0.091 | 0.000 | 0.012 | 9.09e-04 |
| TaVL | Male | Positive | 4189 | 0.215 | -0.001 | 0.026 | 1.01e-25 |
| TaVR | Female | Negative | 3609 | -0.475 | 0.002 | 0.084 | 3.73e-71 |
| TaVR | Female | Positive | 1 | NA | NA | NA | NA |
| TaVR | Male | Negative | 5130 | -0.519 | 0.002 | 0.058 | 7.99e-69 |
| TaVR | Male | Positive | 7 | 0.167 | -0.001 | 0.018 | 7.76e-01 |
